# Burnout and Psychological Wellbeing Among Psychotherapists: A Systematic Review

**DOI:** 10.3389/fpsyg.2022.928191

**Published:** 2022-08-15

**Authors:** Angelika Van Hoy, Marcin Rzeszutek

**Affiliations:** Faculty of Psychology, University of Warsaw, Warsaw, Poland

**Keywords:** burnout, wellbeing, distress, psychotherapy, systematic review

## Abstract

**Objectives:**

The general aim of this systematic review is to synthesize, analyze, and critically review existing studies concerning the relationship between sociodemographic, intrapersonal, and work-related factors and burnout, as well as psychological wellbeing among psychotherapists.

**Methodology:**

We performed a structured literature search utilizing the PRISMA framework in the following databases: Web of Science, Scopus, MedLine, PsyARTICLES, ProQuest, and Google Scholar. The most relevant inclusion criteria were quantitative and peer-reviewed articles published in English.

**Results:**

After the selection process, we accepted 52 articles for further systematic review. Thirty-eight studies examined burnout among psychotherapists, while the other 14 studies focused on psychological wellbeing in this sample.

**Conclusions:**

Burnout and wellbeing among psychotherapists are related to numerous sociodemographic (e.g., age, gender), intrapersonal (e.g., coping, personality), and work-related characteristics, including work settings and professional support in this profession (e.g., supervision or personal therapy). However, the high heterogeneity observed between studies in terms of burnout and wellbeing operationalization and measurement warrants more consistent and advanced methodological models of these theoretical constructs in the future in this specific sample.

## Introduction

Although Freud (1937, 1964) highlighted several negative phenomena in therapeutic settings constituting the *danger of analysis* for the analysts themselves, studies on the psychological health of psychotherapists are lacking in psychotherapy, which has traditionally concentrated solely on the clients of psychotherapy rather than on the psychotherapists (see systematic reviews: Simionato and Simpson, 2018; Lee et al., 2020). Nevertheless, the psychotherapeutic occupation is associated with multidimensional psychological distress, primarily including a high degree of emotional strain and constant demands for empathy, which all pose a significant risk of burnout among psychotherapists (Simionato and Simpson, 2018). Maslach et al. (2001) created one of the most prevalent in the literature model of burnout, which defines this syndrome in terms of emotional exhaustion, depersonalization, and reduced feelings of work-related personal accomplishment. According to Maslach et al. (2001), burnout is particularly common in helping professions. However, it is worth underlining the fact that in contrast to other helping occupations in the health care field (e.g., physicians or nurses; Schaufeli et al., 2009; Woo et al., 2020), burnout research among psychotherapists is much less prevalent (Lee et al., 2020). However, existing studies have demonstrated that psychotherapists who experience burnout, are no longer able to manage therapeutic processes, which may even endanger their clients (e.g., Farber and Heifetz, 1982; Ackerley et al., 1988; Rupert and Morgan, 2005; Berjot et al., 2013). They also suffer from several somatic and psychological disorders, including back pain, headaches, gastroenteritis, depression, and substance abuse (Raquepaw and Miller, 1989; Rupert and Kent, 2007), and they frequently express job turnover intentions (Rosenberg and Pace, 2006; Garcia et al., 2014). Previous systematic reviews on burnout among psychotherapists (Simionato and Simpson, 2018; Lee et al., 2020) have concentrated almost entirely on homogeneous groups of predictors (work-related or sociodemographic factors, e.g., caseload, years of experience, age, or gender) and the burnout measures used (mostly Maslach Burnout Inventory and its scales: emotional exhaustion, depersonalization, and reduced personal accomplishment; Maslach et al., 2001). Nevertheless, it is not entirely clear why there are so many discrepancies in burnout prevalence among psychotherapists from various countries, ranging from 6% to as high as 54% (Farber, 1990; Hannigan et al., 2004; Berjot et al., 2017). On the one hand, this may be related to overlooking other burnout measures in this occupational group and alternative theoretical burnout models (e.g., Demerouti et al., 2001). On the other hand, an insufficient focus on psychotherapists' intrapersonal variables (personality, social support, and self-compassion) as burnout risk factors may also be a reason for the aforementioned inconclusive findings on burnout prevalence in this professional group (Rzeszutek and Schier, 2014; Yip et al., 2017).

Compared to burnout studies, much less research has investigated the issue of psychological wellbeing among psychotherapists (Laverdière et al., 2018, 2019; Brugnera et al., 2020). In other words, while there is a relative consensus regarding why and how the psychotherapeutic profession may be damaging to psychotherapists' mental health, very little is known about personal, social, and work-related characteristics that can foster higher wellbeing and quality of life among psychotherapists. To the best of our knowledge, the existing literature does not contain a systematic review on that topic. This latter issue is of fundamental significance, as several classic reviews have noted that clients choose to work with psychotherapists who they perceive as psychologically healthy and satisfied with their own life (Wogan and Norcross, 1985; Lambert and Barley, 2001). In addition, some authors have observed that the poor quality of life and associated mental difficulties of a psychotherapist may significantly hamper their ability to forge a therapeutic alliance (Enochs and Etzbach, 2004; Holmqvist and Jeanneau, 2006). Overall, the aforementioned results support a need for further studies on psychotherapists' mental health, both from a negative (burnout), as well as a positive perspective (wellbeing) as a crucial issue related to the overall process and outcomes of psychotherapy.

## Objective

The general aim of this systematic review is to synthesize, analyze, and critically review existing studies concerning the relationship between intrapersonal and work-related factors and burnout and psychological wellbeing among psychotherapists. We focused on various theoretical models and associated measures of burnout in the literature (see Methodology). Regarding the concept of wellbeing, we concentrated on its vast operationalizations and assessments, as suggested in the respective literature (Laverdière et al., 2018, 2019; Brugnera et al., 2020), to better capture the uniqueness of that phenomenon in that specific occupation. More specifically, we included both positive (e.g., quality of life, satisfaction with life, and satisfaction with job) and negative dimensions of wellbeing among psychotherapists (e.g., depression, traumatic stress, and secondary traumatic stress). Importantly, we wanted to clearly distinguish these latter factors in particular, namely negative wellbeing indicators for burnout, based on numerous authors indicating that they are two robust and separate constructs (see e.g., Bakker et al., 2000; Maslach et al., 2001; Schaufeli et al., 2009; Koutsimani et al., 2019).

## Methodology

### Systematic Review Protocol

**Figure 1:**
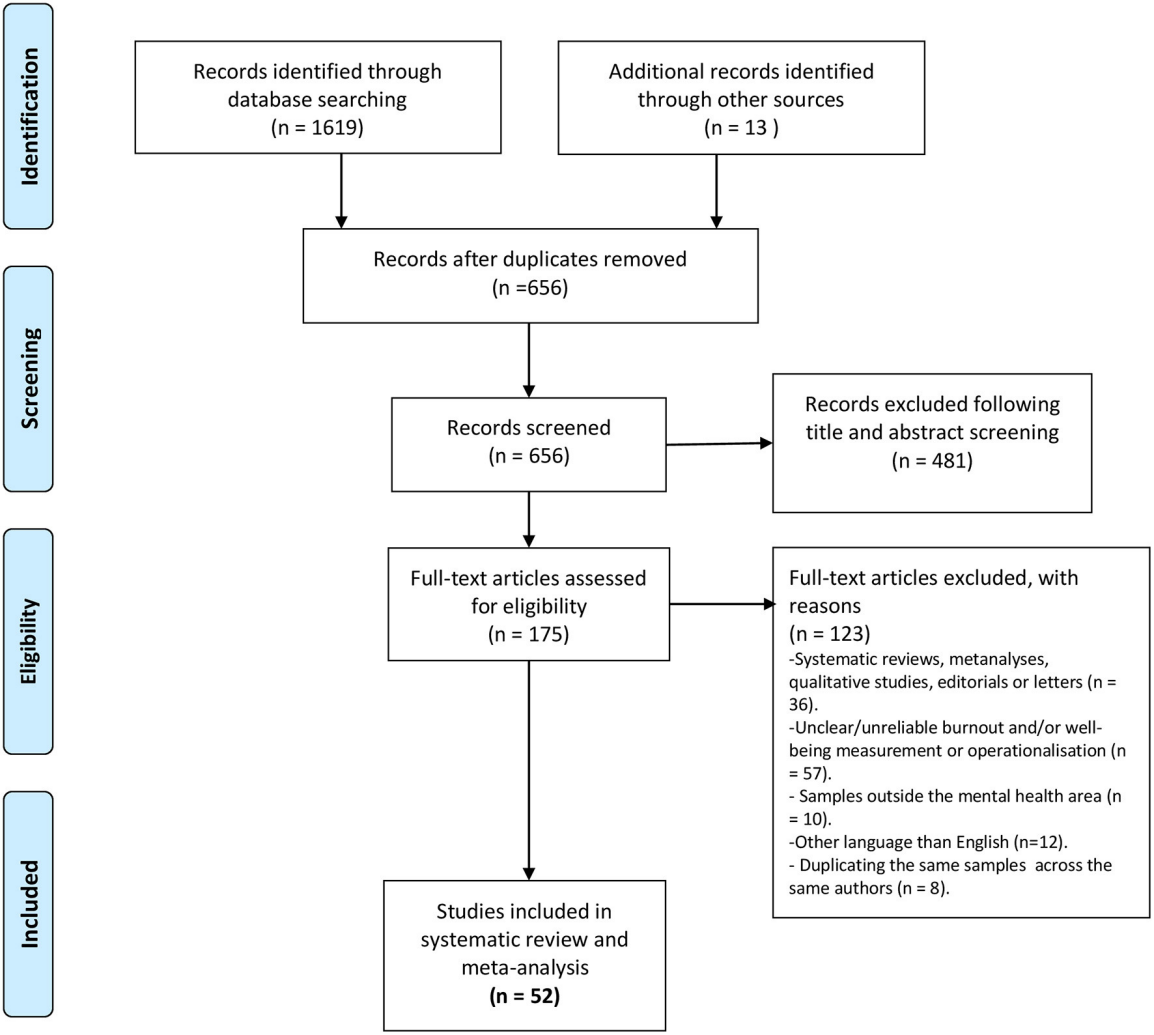
Flow diagram. Based on: Moher et al. (2009).

The two authors of this review have performed the literature search and review, which adhered to the standards of the Preferred Reporting Items for Systematic Reviews and Meta-analyses (PRISMA) statement (Moher et al., 2009; see Figure 1). We then searched the following databases on 06 November 2021: Web of Science, MedLine, Scopus, ProQuest, and PsyARTICLE. We also focused on Google Scholar as an additional source of *gray literature* (Bellefontaine and Lee, 2014). In Boolean algebra, the query consisted of the following terms for burnout: (“burnout” OR “fatigue” OR “job strain” OR “job stress^*^” OR “exhaustion” OR “occupational stress” OR “pressure” OR “cop^*^” OR “manag^*^”) AND (“psychologist^*^” OR “psychotherap^*^” OR “mental health professional^*^” OR “mental health work^*^” OR “social worker^*^” OR “counsel^*^”*)*. The respective search terms for wellbeing were as follows: (“wellbeing” OR (“well” AND “being”) OR (“life” AND “satisf^*^”) OR “life-satisf^*^” OR “wellness” OR (“life” AND “quality”) OR “life-quality” OR “depress^*^” OR “anxi^*^” OR (“post-traumatic” AND “stress”) OR (“posttraumatic” AND “stress”) OR “ptsd”) AND (“psychologist^*^” OR “psychotherap^*^” OR “mental health professional^*^” OR “mental health work^*^” OR “social worker^*^” OR “counsel^*^”*)*. In this review, we used the Covidence software to screen references and undertake data extraction.

### Study Selection Criteria

Despite the English-language criterion, eligible studies must also meet the requirements enumerated below to be included in the systematic review:

(1) Type of study: We included only peer-reviewed, quantitative, empirical articles that assessed the relationship between work-related, personal, and social factors and burnout, as well as psychological wellbeing among psychotherapists. We eliminated other systematic reviews and meta-analyses, as well as editorials, letters, and qualitative studies.

(2) Participants: We included studies with samples of psychotherapists, with no restrictions related to gender, age, ethnicity, work experience, or therapeutic modality. We also accepted studies with mixed representations of, broadly speaking, mental health workers, which consisted of psychotherapists, as well as psychologists, psychiatrists, coaches, etc. We excluded studies that dealt with burnout or wellbeing among health care workers outside of the mental health arena.

(3) Methodology: We only accepted studies with psychometrically sound measurements of burnout and wellbeing outcomes. We excluded studies with no psychometric burnout or wellbeing measures.

(4) Quality of study: We based this on the Quality Assessment Tool for Observational Cohort and Cross-Sectional Studies (Shuang et al., 2015), which consisted of 14 criteria. Two independent evaluators investigated the studies (see Results and Figure 1). The evaluators were particularly interested in whether the study utilized validated measures using psychometric data and presented a clear operationalization of burnout and wellbeing and their predictors. Moreover, if we encountered articles by the same authors, we determined whether they used an identical sample of participants more than once. If this happened, only one of them was included in the final analysis.

## Results

### Screening and Eligibility

Initially, we identified 1,632 titles and abstracts *via* the search of the electronic databases, including 479 hits on Web of Science, 262 hits on MedLine, 506 hits on SCOPUS, 352 hits on Proquest, 20 hits on PsyARTICLES, and 13 hits on Google Scholar. After removing duplicates, we pared them down to 656 potentially eligible articles for further screening. After a comparison of two independent coders, including the consistency of abstract and full-text screening, as well as the quality assessment, 175 full articles remained for the assessment. Using the exclusion criteria, we eliminated 123 papers. Ultimately, we accepted 52 articles for systematic review, 38 for studies on burnout, and 14 for studies on psychological wellbeing among psychotherapists.

We managed to find articles dating from 1986 through 2021. The total sample size was n = 15,229, including 3,972 men, 11,005 women, 18 participants, who declared the “other” option, and 234 participants in a study that did not specify gender. Finally, 49/52 (94%) of the analyzed studies were cross-sectional.

### Burnout and Wellbeing Measures

The most common measure of burnout was the Maslach Burnout Inventory (MBI, Maslach and Jackson, 1986), which measures three dimensions of burnout (emotional exhaustion, depersonalization, and lack of personal accomplishment). Some authors using this scale created the global burnout score (see Table 1). Less utilized burnout measures were as follows: the Copenhagen Burnout Inventory (Kristensen et al., 2005), Oldenburg Burnout Inventory (Demerouti et al., 2001), and Professional Quality of Life Scale (ProQOL; Stamm, 2010). These all followed the global burnout scores. The most frequently used wellbeing measures were Satisfaction with Life (Satisfaction with Life Scale; Diener et al., 1985), Professional Quality of Life Scale (Stamm, 2010), Job Satisfaction Survey (Spector, 1985), and Psychological Wellbeing Scales (Ryff, 1989).

**Table 1:** Summary of data on burnout among psychotherapists.

**References**	**Year & country**	**Study design**	**Burnout measure**	**Burnout predictors**	**Sample (gender)**	**Sample (mean age in years)**	**Stable relationship (% of sample)**	**Workload hours week / experience years**	**Work setting (% of public sector)**	**Supervision/ personal therapy**	**Therapeutic modality**	**Main conclusions**
1. Kahill (1986)	1986, USA	Cross-sectional	“Tedium” burnout measure	Social Support, Professional Expectations	• M – 127 • F – 128 • Total – 255	36,2	71 %	33,2 / 11,1	100%	N/A	CBT – 100%	Social support and professional expectations about the job were negatively related to burnout among psychotherapists. Burnout was not associated with professional experience or to other demographic factors in this sample of psychotherapists.
2. Ackerley et al. (1988)	1988, USA	Cross-sectional	Maslach Burnout Inventory	Age, Overinvolvement, Lack of control of therapy setting, Medical issues, Sexual Abuse, Sexual Dysfunction	• M – 410 • F – 152 • Total – 562	44,15	79%	39,22 / 13,8	39%	Yes / yes	• PD – 20 % • CBT – 9% • Hum – 6% • Int - 56% • Syst – 9%	Significant burnout predictors in this sample were: younger age, lack of control in the therapeutic setting, feeling overcommitted to clients, problems with physical health, history of sexual abuse and sexual dysfunctions. It was also found that lack of personal psychotherapy and lack of supervision correlated positively with burnout intensity.
3. Huberty and Huebner (1988)	1988, USA	Cross-sectional	Maslach Burnout Inventory	Job & Role definitions Time pressures (heavy workload) External pressures (superior pressure) Internal pressures (personality conflicts) Age	• M – N/A • F – N/A • Total - 234	38,72	N/A	N/A / 7,54	N/A	N/A	CBT – 100%	Role definitions, time pressure (heavy workload), external and internal pressures were all related to burnout among psychotherapists. Younger psychotherapists declared higher burnout level.
4. Raquepaw and Miller (1989)	1989, USA	Cross-sectional	Maslach Burnout Inventory	Work load Work setting (public)	• M – 26 • F – 42 • Total - 68	N/A	N/A	N/A	N/A	N/A	Syst – 100%	Working for a public agency and perceived caseload were the strongest predictors of burnout among psychotherapists. Symptoms of urnout were related to intention to leave this job for other professions.
5. van der Ploeg et al. (1990)	1990 Denmark	Cross-sectional	Maslach Burnout Inventory	Age Work experience	• M−69 • F – 29 • Total – 98	36,2	N/A	32,1/11,1	100%	N/A	CBT – 100%	Younger age, less experience in psychotherapy and working in a public sector (compared to private practice) were the strongest predictors of burnout among psychotherapists.
6. Mills and Huebner (1998)	1998, USA	Longitudinal	Maslach Burnout Inventory	Work experience, Perceived job stress, Neuroticism, Extraversion, Agreeableness, Conscientiousness	• M – 60 • F – 165 • Total – 225	40,3	N/A	N/A / 10,4	100%	N/A	Int – 100%	Less experience in psychotherapy, high level of perceived stress in job as well as personality traits (neuroticism positively, extraversion, agreeableness and conscientiousness negatively) were the most significant burnout predictors among psychotherapists.
7. Wilkerson and Bellini (2006)	2006, USA	Cross-sectional	Maslach Burnout Inventory	Emotion-Oriented Coping	• M – 22 • F – 56 • Total – 78	43,19	64,1%	N/A / 11	90%	N/A	Int – 100%	Emotion-oriented coping style was the strongest burnout predictor among psychotherapists.
8. Wiseman and Egozi (2006)	2007, Israel	Cross-sectional	Burnout Questionnaire	Personal Therapy	• M – 16 • F – 83 • O – 4 • Total – 103	41,2	N/A	N/A / 10,6	100%	Yes / yes	• CBT – 50% • Int – 50%	Personal therapy occurred to be the strongest buffer against burnout symptoms among psychotherapists.
9. Ben-zur and Michael (2007)	2007, Israel	Cross-sectional	Maslach Burnout Inventory	Social Support, Problem-oriented coping	• M – 0 • F – 249 • Total – 249	41,66	73%	37,62 / N/A	100%	N/A	CBT – 100%	Appropriate social support at work as well as problem-focused coping with stress were the most important buffers against burnout among psychotherapists.
10. Deighton et al. (2007)	2007, Germany, Austria, Switzerland	Cross- sectional	Maslach Burnout Inventory	Supervision in working with trauma clients Prevalence of psychotherapists own trauma history	• M – 34 • F – 65 • O – 1 • Total – 100	N/A	N/A	24,7 / 7,7	100%	N/A	• PD – 17% • CBT – 14% • Hum – 8% • Int – 31% • Syst – 12%	Supervision and a low prevalence of psychotherapists own trauma history were related to lower burnout level among trauma psychotherapists.
11. Rupert and Kent (2007)	2007; USA	Cross-sectional	Maslach Burnout Inventory	Age Work load Administrative paperwork Negative client behaviors Overinvolvement Sense of control over work	• M – 248 • F – 347 • Total – 595	51,98	75%	39,19/17,19	42%	Yes, yes	• PD – 23 % • CBT – 32% • Hum – 0% • Int - 20% • Syst – 25%	The main important burnout risk factors among psychotherapists were: younger age, too much workload, negative client behaviors and work settings (higher burnout in public sector, with less subjective control over the therapeutic work). In addition it was found that females declared less burnout symptoms than males.
12. Rupert et al. (2009)	2009, USA	Cross-sectional	Maslach Burnout Inventory	Work-Family conflict, family-work conflict, workload, Sense of control over work, Family support	• M – 205 • F – 292 • Total – 497	54,1	96%	35,2 / 19,6	39%	Yes / yes	• CBT – 30% • Int – 30% • Syst – 40%	The strongest predictors of burnout among psychotherapist were perceived conflicts on the dimensions work-family or family-work, as well as high workload. Buffers against burnout were family support and perceived sense of control over the job.
13. Kim and Lee (2009)	2009, South Korea	Cross-sectional	Maslach Burnout Inventory	Perceived job stress, Supervision	• M – 110 • F – 101 • Total – 211	42,2	N/A	N/A	100%	Yes / N/A	Syst – 100%	Perceived job stress was positively related with burnout level among psychotherapists, while support received from supervision buffered against this syndrome in this sample of psychotherapists.
14. Emery et al. (2009)	2009, Australia	Cross-sectional	Maslach Burnout Inventory	Gender, Work setting, Personal resources, Sense of control over work	• M – 54 • F – 136 • Total – 190	34,5	75,3%	N/A / 7,1	45%	N/A	CBT – 100%	Significant burnout risk factors included: gender (females, but only when they work in public sector), public work sector, lack of personal resources and lack of feeling of control over the work.
15. D'Souza et al. (2011)	2011, Australia	Cross-sectional	Copenhagen Burnout Inventory	Perceived job stress Perfectionism	• M – 12 • F – 75 • Total – 87	45,6	N/A	N/A, 3,5	95%	N/A	CBT – 100%	There was a significant positive relationship between burnout level and the intensity of perfectionism and perceived stress at work.
16. Kim et al. (2011)	2011, USA	Longitudinal	Maslach Burnout Inventory	Physical health	• M – 62 • F – 223 • Total – 285	46,1	N/A	N/A / 18,1	100%	N/A	CBT – 100%	Burnout symptoms predicted deterioration in psychotherapists' physical health (see headaches, gastrointestinal problems, respiratory infections) over three years period of observation.
17. Acker (2012)	2011, USA	Cross-sectional	Maslach Burnout Inventory	Perceived job stress	• M – 123 • F – 337 • Total – 460	41	57%	N/A / 11	100%	N/A	Int – 100%	Perceived job stress occurred to be the strongest burnout predictor among psychotherapists.
18. Malinowski (2013)	2013, USA	Cross-sectional	Maslach Burnout Inventory	Humor oriented coping	• M – 42 • F – 91 • Total – 133	53,5	N/A	26,1 / 19,5	54%	N/A	CBT – 100%	Self-enhancing humor, as coping style, was positively related with the level of perceived personal accomplishment among psychotherapists.
19. di Benedetto and Swadling (2014)	2014, Australia	Cross- sectional	Copenhagen Burnout Inventory	Mindfulness Work experience Career-sustaining behaviors	• M – 22 • F – 145 • Total – 167	42,47	77,8	N/A	80%	N/A	N/A	Practicing mindfulness and more years of experience seemed to protect from burnout among Australian psychotherapists.
20. Rzeszutek and Schier (2014)	2014, Poland	Cross-sectional	Oldenburg Burnout Inventory	Social Support, Briskness, Perseveration	• M – 89 • F – 111 • Total – 200	35,94	N/A	26,1 / 19,5	60%	Yes / yes	• CBT – 50% • Hum – 50%	The level of burnout symptoms among psychotherapists was positively related to perseveration and negatively linked to briskness and perceived social support.
21. Steel et al. (2015)	2015, UK	Cross-sectional	Maslach Burnout Inventory	Work demands Sense of control over work Perceived job stress	• M - 20 • F – 74 • Total – 94	36,9	N/A	N/A,1,9	100%	N/A	• CBT – 88% • Int – 12%	Significant predictors of burnout among psychotherapists were high work demands, perceived job stress and lack of control over the organization of work.
22. Rasmussen et al. (2016)	2016, Australia	Cross-sectional	Maslach Burnout Inventory	Age, Work demands, Work reward, Overinvolvement, Meaningfulness of work	• M – 66 • F – 351 • Total – 417	49,5	N/A	34,9 / 5,5	82%	Yes/ N/A	• CBT – 52% • Int – 17% • Syst – 31%	Significant predictors of burnout among psychotherapists were younger age, perceived high work demands, perceived low work efforts, over- involvement in therapeutic process and lack of sense of meaning in work.
23. Kim (2017)	2017, South Korea	Cross-sectional	Maslach Burnout Inventory	Work experience, Personal resources, Secondary Traumatic Stress, Workload	• M – 59 • F – 120 • Total – 179	32,4	N/A	16,2 / 1,75	100%	N/A	• Int – 18% • Syst – 82%	Significant predictors of burnout among psychotherapists were little work experience, high work load, few personal resources, perceived high work demands, and high symptoms of secondary traumatic stress.
24. Berjot et al. (2017)	2017, France	Cross- sectional	Maslach Burnout Inventory	Work setting Type of work contract Age Seniority in workplace	• M – 66 • F – 598 • Total – 664	35,44	N/A	N/A	54%	N/A	N/A	Working in a company, having multiple work contracts, younger age and seniority in the workplace all showed to be significant predictors of burnout among psychotherapists.
25. Garcia et al. (2018)	2018, USA	Cross-sectional	Maslach Burnout Inventory	Rule overload in work Vulnerability to oversight Politics influence on treatment methods Supervision	• M – 143 • F – 338 • Total – 481	41,2	N/A	N/A	100%	N/A	• CBT – 69% • Int – 16% • Syst – 15%	Burnout was particularly associated with reports of “political influence” on treatment, feelings of vulnerability of complaints to leadership or government, and rule overload in a sample of trauma psychotherapists. Clinical supervision buffered the burnout symptoms in this sample.
26. Simpson et al. (2019)	2019, Australia	Cross-sectional	Maslach Burnout Inventory	Work demands, Abandonment, Mistrust/Abuse, Emotional Inhibition, Detached Protector	• M – 87 • F – 356 • Total – 443	42,93	52,8%	N/A	54%	N/A	• PD – 7% • CBT – 68% • Int – 17% • Syst – 8%	Job demands, early maladaptive schemas and maladaptive coping modes significantly predicted burnout among psychotherapists.
27. Lee et al. (2019)	2019, South Korea	Cross-sectional	Maslach Burnout Inventory	Perceived job stress, Resilience	• M – 56 • F – 214 • Total – 270	35,5	52%	N/A	100%	N/A	N/A	It was found that the level of perceived stress at work positively, and resilience negatively were associated with burnout among psychotherapists.
28. von Hippel et al. (2019)	2019, Australia	Cross-sectional	Copenhagen Burnout Inventory	Work satisfaction, Commitment to organization, Commitment to profession, Work engagement, Workplace wellbeing, Intentions to leave organization, Intentions to leave profession	• M – 80 • F – 265 • O – 4 • Total – 349	35	N/A	N/A / 5,65	100%	N/A	N/A	Burnout was significantly related to lower job satisfaction and lower job engagement, decreased workplace well-being, and increased turnover rates among psychotherapists.
29. George-Levi et al. (2020)	2020 Israel	Cross-sectional	Maslach Burnout Inventory	Sense of coherence	• M – 26 • F – 78 • Total – 104	37,4	N/A	N/A6,5	100%	N/A	CBT – 100%	Sense of coherence buffered the burnout symptoms among psychotherapists and the perceived loneliness moderated this association.
30. Tsai et al. (2020)	2020, USA	Cross-sectional	Maslach Burnout Inventory	Age, Ethnicity (Caucasian)	• M – 16 • F – 26 • Total – 42	39,6	36%	42,9 / 4,7	100%	Yes / N/A	CBT – 100%	Significant predictors of burnout among psychotherapists were younger age and being white.
31. Hricová (2020)	2020, Slovakia	Cross-sectional	Maslach Burnout Inventory	Perceived job stress, Self-care	• M – 80 • F – 618 • Total - 698	43,9	N/A	20,34 / 13,12	100%	Yes / N/A	• CBT – 32% • Int – 41% • Syst – 27%	There was a significant association between perceived job stress and burnout and this relationship was also mediated by health self-care among psychotherapists.
32. Allwood et al. (2020)	2020, Sweden	Cross-sectional	Shirom-Melamed Burnout Questionnaire	Age, Ruminations: general, Ruminations: work, Family-work conflict, Work demands	• M – 182 • F – 646 • Total – 828	42,1	N/A	38 / N/A	100%	N/A	CBT – 100%	Burnout was significantly associated with younger age, tendency to ruminations (at work and in general) perceived work conflicts as well as high job demands among psychotherapists. Moreover, results showed that women experienced higher burnout levels than men.
33. Kotera et al. (2021)	2021, UK	Cross-sectional	Maslach Burnout Inventory	Age Workload Work-life balance	• M – 23 • F – 83 • Total – 106	47,42	N/A	31,3/9,3	100%	N/A	• Int – 50% • Syst – 50%	Younger age, high workload and problems with work-life balance were found to significantly predict burnout among psychotherapists.
34. Zarzycka et al. (2021)	2021, Poland	Cross-sectional	Link Burnout Questionnaire	Therapeutic relationship: relational depth Therapeutic relationship: relational quality Psychological wellbeing	• M – 75 • F – 26 • Total – 101	44,34	56%	N/A / 10	19%	Yes / yes	Hum – 100%	Aspects of therapeutic relationship (relational depth and relational quality) were the strongest buffers against burnout among psychotherapists. Burnout symptoms significantly hampered wellbeing of psychotherapists.
35. Chang and Shin (2021)	2021, South Korea	Cross-sectional	Professional Quality of Life Scale	Compassion satisfaction, Compassion fatigue, Adaptive emotion regulation, Maladaptive emption regulation, Perceived job stress, Negative client behaviors	• M – 45 • F – 80 • Total – 125	N/A	N/A	N/A	100%	N/A	N/A	Burnout was positively related with compassion fatigue level, maladaptive emotion regulation, and experience of aggression by clients among psychotherapists. Conversely, compassion satisfaction and adaptive emotion regulation strategies buffered from symptoms of burnout in this sample.
36. Smout et al. (2021)	2021, Australia	Cross-sectional	Maslach Burnout Inventory	Resilience Work demands Coping style: detached protector	• M – 82 • F – 343 • Total – 425	42,79	53%	N/A	54%	N/A	• CBT – 89% • Int – 11%	Maladaptive coping (detached protector coping mode), high work demand seemed to be significantly associated with burnout among psychotherapists. Resilience acted as a buffer against burnout symptoms in this sample.
37. McCade et al. (2021)	2021, Australia	Cross-sectional	Copenhagen Burnout Inventory	Depression, Self-Compassion	• M – 44 • F – 203 • O – 1 • Total – 248	41,04	75%	N/A / 12,2	82%	N/A	• CBT – 50% • Int – 25% • Syst – 25%	Self-compassion may be treated as protective facto against burnout and depression among psychotherapists.
38. Litam et al. (2021)	2021, USA	Cross-sectional	Professional Quality of Life Scale	COVID-19 related distress Resilience Compassion fatigue	• M – 24 • F −135 • O – 2 • Total – 161	39,1	N/A	N/A	68%	1, N/A	• CBT – 50% • Int – 50%	COVID-19 related distress and high level of compassion fatigue were the strongest predictors of burnout among psychotherapists. Resilience acted as a buffer against burnout symptoms in this sample.

*Gender: M, Male; F, Female; O, Other; Therapeutic Modality: PD, Psychodynamic; CBT, Cognitive Behavioral Therapy; Hum, Humanistic; Int, Integrative; Syst, Systemic; N/A, Not Available*.

It is worth mentioning several remarks concerning burnout and wellbeing assessment and operationalization. First, the vast majority of studies adopted the Maslach et al. (2001) burnout model. Second, in the eligible studies, the authors typically performed statistical analysis using the global burnout/wellbeing score. As the majority of studies used such a global burnout and/or wellbeing score, the final conclusions were also drawn from such global indicators. If more than one dimension of burnout or wellbeing was mentioned in the study, the result with the highest strength of associations with appropriate predictors was selected, which was recommended by other authors as well (Simionato and Simpson, 2018).

Tables 1, 2 summarize all the details related to the systematic review of our 52 final studies related to burnout (*n* = 38) and psychological wellbeing (*n* = 14) among psychotherapists.

**Table 2:** Summary of data on wellbeing among psychotherapists.

**Authors**	**Year and country**	**Study design**	**Wellbeing outcome and measure**	**Wellbeing predictors**	**Sample (gender)**	**Sample (mean age in years)**	**Stable relation (% of sample)**	**Workload hours week / experience years**	**Work setting (% of public sector)**	**Supervision/ personal therapy**	**Therapeutic modality**	**Main conclusions**
1. Schlarb et al. (2012)	2012, Germany	Cross-sectional	Satisfaction with life (Satisfaction with Life Scale)	Workload, Job demands, Insomnia level	• M – 193 • F – 581 • Total - 774	46,1	N/A	39,7/17,5	22%	N/A	• PD – 40% • CBT – 40% • Int – 10% • Syst – 10%	Almost 45% of the studied psycho therapists suffered from insomnia symptoms. Workload, specific job demands and insomnia level were the strongest negative predictors of life satisfaction among psychotherapists.
2. Puig et al. (2012)	2012, USA	Cross-sectional	Wellness (The Five Factor Wellness Inventory)	Devaluing client, Deterioration in Personal life, Incompetence, Exhaustion	• M – 23 • F – 106 • Total – 129	40,67	N/A	N/A	89%	N/A	• Int – 53% • Syst – 47%	Significant predictors of wellness among psychotherapists were specific behaviors of clients (devaluing client), problems in personal life, subjective feeling of incompetence and exhaustion by the work.
3. Hardiman and Simmonds (2013)	2013, Australia	Cross-sectional	Existential wellbeing (Spiritual Wellbeing Scale)	Severity of client trauma, Emotional exhaustion	• M – 18 • F – 71 • Total – 89	49,69	N/A	20,48/16,4	21%	N/A	• PD – 17% • CBT – 25% • Hum – 4% • Int – 48% • Syst – 6%	Psychotherapists declared high level of existential wellbeing coped better with highly traumatized clients and avoided emotional exhaustion at work.
4. Rzeszutek et al. (2015)	2015, Poland	Cross-sectional	Secondary traumatic stress (PTSD Questionnaire: Factorial Version)	Temperament: Emotional reactivity, Temperament: Sensory Sensitivity, Social Support	• M – 21 • F – 59 • Total – 80	39,48	N/A	10,44/9,45	66%	Yes / N/A	• PD – 11% • CBT – 44% • Hum – 36% • Int – 4% • Syst – 5%	The level of secondary traumatic stress symptoms among trauma psychotherapists was positively related to emotional reactivity and negatively linked to sensory sensitivity and perceived social support.
5. Roncalli and Byrne (2016)	2016, Ireland	Cross-sectional	Job satisfaction (Minnesota Satisfaction Questionnaire)	Workload, Work experience, Supervision, Team work with co-workers Satisfaction with work with co-workers	• M – 18 • F – 59 • Total - 77	37,8	N/A	11,3/6,7	100%	Yes/ N/A	• CBT – 100%	Supervision and satisfaction with teamwork with colleagues occurred to be the strongest predictors of psychotherapists' job satisfaction.
6. Yip et al. (2017)	2017, China	Cross-sectional	Compassion fatigue (The Professional Quality of Life Scale)	Mindfulness, Self-Compassion: self-warmth, Self-Compassion: Self-coldness	• M – 9 • F – 68 • Total – 77	35,2	N/A	N/A /4,5	95%	N/A	CBT – 100%	The relationship between mindfulness and compassion fatigue was mediated by self-coldness (negative qualities in self-compassion). Thus, mindfulness may buffer the compassion fatigue among psychotherapists, but self-compassion of therapists matters.
7. Fleury et al. (2017)	2017, Canada	Cross-sectional	Job satisfaction (Job Satisfaction Survey)	Team work with co-workers Interdisciplinary collabaration	• M – 15 • F – 53 • Total – 68	40	N/A	22,45/6,1	72%	Yes / N/A	Int – 100%	Team work, especially mutual supervision and the frequency of interdisciplinary collaboration with colleagues representing other psychotherapy organizations occurred to be the strongest predictors of psychotherapists' job satisfaction.
8. Hitge and van Schalkwyk (2018)	2017, South Africa	Cross-sectional	Psychological wellbeing (Mental Health Continuum Short Form)	Meaningfulness, Resilience	• M – 72 • F – 182 • Total – 254	43,2	N/A	7,5/13,4	53%	Yes / N/A	N/A	Searching for meaning and resilience as a personality trait were the strongest predictors of psychotherapist's wellbeing.
9. Laverdière et al. (2018)	2018, Canada	Cross-sectional	Satisfaction with Life (Satisfaction with Life Scale)	Perceived stress, Work experience, Workload	• M – 53 • F – 187 • Total – 240	42,25	N/A	23,5/13,5	60%	Yes / N/A	• PD−31% • CBT – 31% • Hum – 15% • Int – 22% • Syst – 1%	Perceived stress, high workload and less years of experience were predictors of poor life satisfaction among psychotherapists.
10. Rupert and Dorociak (2019)	2019, USA	Cross-sectional	Satisfaction with Life (Satisfaction with Life Scale)	Self-Care, Perceived job stress	• M – 127 • F – 295 • Total – 422	50,48	76%	44,13/16,71	66%	Yes/ N/A	N/A	Self-care enhanced psychotherapists' wellbeing and the main mechanism in that process was reducing the level of perceived stress at work.
11. Yela et al. (2020)	2019 Spain	Longitudina;	Psychological wellbeing (Psychological Wellbeing Scales)	Mindfullness Self-compassion	• M – 7 • F – 54 • Total – 61	25,6	N/A	11,3/1,1	100%	N/A	N/A	Training in mindfulness and self-compassion showed significant improvement in psychotherapist' wellbeing over time.
12. Müller et al. (2020)	2019, Germany	Cross-sectional	Satisfaction with Life (Life Satisfaction Questionnaire)	Work-related strain, Supervision	• M – 54 • F – 56 • Total – 110	51,4	N/A	41,6/ 13,7	25%	Yes / N/A	Int – 100%	Supervision significantly improved psychotherapists' job satisfaction when they experienced a high amount of work-related strain.
13. Brugnera et al. (2020)	2021, Italy	Cross-sectional	Psychological wellbeing (Psychosocial General Wellbeing Index)	Attachment anxiety, Attachment avoidance, Reflective Functioning, Gender, Age	• M – 84 • F – 332 • Total – 416	43,94	76%	N/A / 10,1	N/A	Yes / N/A	• PD – 13% • CBT – 11% • Int – 66% • Syst – 1%	Attachment anxiety and attachment avoidance were negatively related, while reflective functioning was positively associated with wellbeing among psychotherapists. Older psychotherapists declared higher wellbeing.
14. Summers et al. (2021)	2021, UK	Cross-sectional	Psychological Wellbeing	Depressive symptoms Harassment or bullying Work experience Age	• M – 320 • F – 1334 • Total – 1654	46,2	N/A	N/A / 4,3	96%	Yes/ N/A	• CBT – 9% • Int – 52% • Syst – 39%	The strongest, negative predictors of psychotherapists' wellbeing were: being harassed, feeling depressed, older age and higher work experience.

*Gender: M, Male; F, Female; O, Other; Therapeutic Modality: PD, Psychodynamic; CBT, Cognitive Behavioral Therapy; Hum, Humanistic; Int, Integrative; Syst, Systemic; N/A, Not Available*.

## Summary of Findings and Main Conclusions

The primary goal of this systematic review was to synthesize, analyze, and critically review already-existing research regarding the relationship between intrapersonal and work-related factors, burnout, and psychological wellbeing among psychotherapists. In terms of burnout, the main focus was on various theoretical models and measures that were associated with the construct, although the Maslach et al. (2001) model was the most prevalent (see further). Within that model, we observed that the most common burnout dimension among psychotherapists was emotional exhaustion, which was consistent with previous reviews on that subject (Lee et al., 2020). The concept of wellbeing was examined in relation to its numerous operationalizations and assessments, specifically in the psychotherapeutic occupation. Both positive and negative dimensions of wellbeing were included. However, as was already underlined, we aimed to clearly distinguish these latter negative wellbeing indicators from burnout based on numerous authors indicating that they are two robust and separate constructs (see e.g., Bakker et al., 2000; Maslach et al., 2001; Schaufeli et al., 2009; Koutsimani et al., 2019). Finally, after a careful selection process, we included 52 articles in the review, 38 for burnout and 14 for wellbeing, published between 1986 and 2021. Selected papers satisfied the selection criteria regarding the content and quality of the studies (see Methods).

### Sociodemographic, Intrapersonal, and Work-Related Factors Related to Burnout Among Psychotherapists

When it comes to sociodemographic correlates of burnout among psychotherapists, it appears that, in particular, age and gender play a significant role. Firstly, a total of nine relevant studies (i.e., those which provided data on the age-burnout association) revealed that younger psychotherapists tend to report increased levels of burnout symptoms in comparison to older and more experienced colleagues in that profession (Ackerley et al., 1988; Huberty and Huebner, 1988; van der Ploeg et al., 1990; Rupert and Kent, 2007; Rasmussen et al., 2016; Berjot et al., 2017; Allwood et al., 2020; Tsai et al., 2020; Kotera et al., 2021). This finding is consistent with the previous meta-analysis concerning the aforementioned relationship in various employment settings (Brewer and Shapard, 2004). In our specific context, this is explained by the fact that young psychotherapists often have excessively high and unrealistic expectations about their role in this job, and the subsequent *reality crash* may be a significant burnout catalyst for them (Rupert and Kent, 2007; Rasmussen et al., 2016; Berjot et al., 2017). Secondly, in terms of gender, studies have yielded mixed findings. On the one hand, higher burnout levels have been recorded among women in comparison to men (Emery et al., 2009; Allwood et al., 2020). This is consistent with gender role theory (Eagly, 1987), which suggests that women are more likely to express *negative* feelings (e.g., emotional and physical fatigue) compared to men, who typically conceal these emotions from others, which does not mean that they do not experience them. Thus, this could indicate an artificial overrepresentation of burnout among females, which would correspond with Rupert and Kent (2007), who reported contrasting evidence, with men experiencing more burnout than women in their sample of psychotherapists. A relevant meta-analysis on that topic demonstrated that men and women can experience burnout in different ways. For instance, women score higher on emotional exhaustion, whereas men score higher on depersonalization (Purvanova and Muros, 2010). In addition, it appears that the psychotherapeutic profession may be especially conducive to burnout among men due to gender differences in self-efficacy, which is usually higher among females in helping professions (e.g., Roohani and Iravani, 2020). Gender also moderates the effects of work stress and the association between self-efficacy and health in various professions (Shoji et al., 2016). Future research should also consider the “close-to-zero” gender differences observed in this context, which may explain discrepancies in this area of research, particularly when using the Maslach model (Purvanova and Muros, 2010).

Secondly, eight studies have highlighted the significant role of intrapersonal variables as burnout predictors among psychotherapists, including predominantly personality traits (Mills and Huebner, 1998; Rzeszutek and Schier, 2014; Lee et al., 2019; George-Levi et al., 2020; Smout et al., 2021) and various stress coping styles (Wilkerson and Bellini, 2006; Ben-zur and Michael, 2007; Malinowski, 2013). Generally, traits related to negative emotionality (e.g., neuroticism, emotional reactivity) were burnout predictors, while those associated with emotional stability and high levels of subjectively perceived resources (e.g., resilience) were buffers against this syndrome in this occupation. Similarly, emotion-oriented coping was positively correlated, while problem-focused coping or the use of humor to deal with work-related stress were negatively linked to burnout symptoms in this occupation. Although there are numerous studies on the personality-burnout (Alarcon et al., 2009) and coping-burnout association (Lee et al., 2016), this problem is still highly understudied in this specific occupation and calls for more research, ideally in the prospective methodological framework. Alternatively speaking, one should remember that aside from work-related characteristics, there are substantial intrapersonal factors related to burnout in that occupation, which until now were not sufficiently underlined in the contemporary reviews on that topic (Simionato and Simpson, 2018; Lee et al., 2019).

Finally, concerning work-related covariates, workload, and/or work experience, work settings and supervision/personal therapy exerted relatively homogenous effects on burnout. Six studies have revealed significant associations between high workload and increased levels of burnout (Huberty and Huebner, 1988; Raquepaw and Miller, 1989; Rupert and Kent, 2007; Rupert et al., 2009; Kim, 2017; Kotera et al., 2021). Similarly, four studies have suggested that less experienced psychotherapists were particularly vulnerable to burnout compared to their more experienced colleagues (van der Ploeg et al., 1990; Mills and Huebner, 1998; di Benedetto and Swadling, 2014; Kim, 2017). Specifically, these latter results generally corresponded with the previously mentioned role of the younger age of therapists as a significant burnout predictor in that job. Additionally, working in the public sector seemed to be positively associated with burnout levels, as highlighted by five studies (Raquepaw and Miller, 1989; van der Ploeg et al., 1990; Rupert and Kent, 2007; Emery et al., 2009; Berjot et al., 2017). This finding was usually explained by a lack of control over their own work environment among psychotherapists and an increased level of bureaucracy in such workplaces compared to private psychotherapists' offices, where they may feel that they have more control over when, where, and how they will be working with their clients. Lastly, personal therapy and/or supervision act as a buffer against burnout, as mentioned by five authors (Ackerley et al., 1988; Wiseman and Egozi, 2006; Deighton et al., 2007; Kim and Lee, 2009; Garcia et al., 2018), which is a highly important argument in the ongoing discussion related to self-care behaviors among psychotherapists (Norcross et al., 2008).

### Sociodemographic, Intrapersonal, and Work-Related Factors Behind Psychological Wellbeing Among Psychotherapists

First of all, it is worth mentioning that to the best of our knowledge, no systematic review has investigated the problem of wellbeing and quality of life among psychotherapists in the literature thus far. This latter issue is of fundamental significance, as psychotherapists' poor quality of life and associated mental difficulties among psychotherapists may significantly hamper the entire psychotherapeutic process (Enochs and Etzbach, 2004; Holmqvist and Jeanneau, 2006). However, as may be visible when we compare the number of studies concerning burnout vs. wellbeing among psychotherapists (see Tables 1, 2), studies on the latter issue are particularly scarce, as attention has still focused mainly on negative aspects of psychotherapists' functioning. Thus, although we intended to fill this existing research gap, the empirical evidence on that subject is minimal compared to that concerning burnout among psychotherapists.

In the context of wellbeing among psychotherapists, the only significant relationships with sociodemographic variables were with age, but two studies presented contrasting results (Brugnera et al., 2020; Summers et al., 2021). On the one hand, older psychotherapists appeared to experience higher levels of wellbeing. This is consistent with findings concerning the role of age in the general population, which indicate that older adults tend to be more satisfied with their lives and experience enhanced psychosocial and economic conditions (Ulloa et al., 2013; Steptoe et al., 2015). On the other hand, older age was also a negative predictor of wellbeing; these results may be “time-specific,” as this was one of the few studies conducted during the COVID-19 era among this specific population. Perhaps older psychotherapists experience greater difficulties organizing their work, mainly due to the obligations inherent to online settings compared to their younger counterparts, who are typically much more fluent in new technologies.

Studies on intrapersonal variables related to psychotherapists' wellbeing have confirmed the role of personality as well as self-care behaviors, including self-compassion and mindfulness practices, in maintaining a high quality of life in that profession (Rzeszutek et al., 2015; Yip et al., 2017; Hitge and van Schalkwyk, 2018; Rupert and Dorociak, 2019; Yela et al., 2020). It appears that greater care for the personal emotional balance of psychotherapists is a crucial, but highly understudied issue, both in the empirical field, as well as in relation to psychotherapists' training procedures involving various therapeutic modalities (Laverdière et al., 2018).

Concerning work-related characteristics, only workload, work experience, and professional support were significant. Workload and amount of experience in the profession appeared to be important in terms of psychological wellbeing (Schlarb et al., 2012; Laverdière et al., 2018; Summers et al., 2021). Higher workload was revealed to be a negative predictor of wellbeing, while less experience seemed to be related to lower life satisfaction, especially if both elements were present. This latter finding is consistent with the previously mentioned data and socio-demographic and work-related factors behind burnout in this profession. Moreover, three studies consistently proved that supervision and general support from team coworkers played a positive role in terms of wellbeing (Roncalli and Byrne, 2016; Fleury et al., 2017; Müller et al., 2020). This is another argument in favor of the significance of professional support in that occupation (Norcross et al., 2008).

### Strengths, Limitations and Future Directions

So far, the focus of existing research has focused on the positive (wellbeing) and negative (burnout) aspects of therapists' functioning. This review adds to the literature concerning these issues. Additionally, this brings awareness to the need for implementing education about self-care practices in psychotherapy training programs. These suggestions also extend to current psychotherapists that have already completed their initial training in the profession. Psychotherapists can benefit from maintaining their wellbeing and taking action to decrease risk for burnout. This can also positively affect their clients.

This systematic review is not free of limitations, which should be delineated along with recommendations for potential future research regarding burnout and wellbeing among psychotherapists. First, we excluded studies that were conducted in languages other than English and/or qualitative research, which could be a valuable source of information related to psychotherapists' mental functioning. In the future, it would be interesting to review qualitative studies on psychological functioning among psychotherapists. Burnout is a global phenomenon, thus, future research should include non-English papers, as well.

Second, while preparing this review, we faced several problems related to the operationalization and measurement of burnout and wellbeing. More specifically, the burnout measures were inconsistent across the included studies; some employed the three scales of burnout (see MBI), while other studies only included statistics based on the global burnout score. The Maslach model and the MBI tool dominated most of the reviewed studies, although this model has been the subject of widespread criticism (Demerouti et al., 2001). In the context of this review, one should underline that the use of MBI cutoff values to screen “cases” of burnout is very problematic, as it may be an insufficient way to diagnose the prevalence of burnout in various samples. Another important criticism concerns the fact that the MBI subscales all contain only one-directional items, which may lead to the artificial clustering of factors (Halbesleben and Demerouti, 2005). Analog problems arose related to measures of psychological wellbeing; we found a large variety of measurements and models of this theoretical construct. Furthermore, there was also a high diversity in burnout and wellbeing predictors. The aforementioned two factors make it highly difficult to summarize these findings in the form of a meta-analysis. Future studies should employ a more unitary operationalization and measurement of burnout and/or wellbeing in this specific sample. Regarding burnout, different models from the Maslach model should be more widely accounted for to avoid the criticism of a lack of theoretically driven research in the burnout field (Demerouti et al., 2001). New insights into the Job Demands–Resources theory may point researchers in a promising direction regarding combating these shortcomings (Bakker and de Vries, 2021).

Third, about 95% of the reviewed studies utilized a cross-sectional design. Thus, no cause-and-effect conclusions can be drawn. Further research should also include more longitudinal studies to examine long-term relationships between burnout and wellbeing and their predictors among psychotherapists. This issue is especially crucial in this profession and its outcomes, with it usually being a long-term process (Norcross et al., 2008).

Fourth, the reviewed studies consisted of heterogeneous groups of psychotherapists, representing not only different therapeutic modalities, but sometimes also those outside of the pure psychotherapeutic occupation. This is related to the fact that the psychotherapeutic occupation may be represented by several occupational groups, as different regulations for the psychotherapeutic profession exist in various countries. In the future, it would be wise to focus on more homogeneous samples of psychotherapists; for example, researchers could compare whether and how different psychotherapy modalities differ concerning psychological functioning (Rzeszutek and Schier, 2014).

Finally, more research should be conducted regarding psychotherapists' wellbeing, as we have noticed that there were almost three times more studies concerning the negative aspects of psychotherapists' functioning compared to research focusing on how to maintain high wellbeing and quality of life in this professional group (Laverdière et al., 2018). Studies have shown that clients often choose to attend therapy with psychotherapists who are perceived as psychologically stable and seem satisfied with their personal lives (Wogan and Norcross, 1985; Lambert and Barley, 2001). Low quality of life among psychotherapists can potentially deteriorate therapeutic alliances with clients as well as the entire therapeutic process (Enochs and Etzbach, 2004; Holmqvist and Jeanneau, 2006).

## Conclusions

Our systematic review suggests that burnout and wellbeing among psychotherapists may largely depend on sociodemographic (e.g., age, gender), intrapersonal (e.g. coping, personality), and work-related characteristics, including work settings and professional support in this profession (e.g., supervision or personal therapy). However, future research is required, particularly studies that adopt more advanced methodological models and burnout/wellbeing operationalizations and assessments (Lee et al., 2019). Numerous studies use various definitions of burnout and wellbeing. It would be beneficial to find universal definitions of the terms in order to address the vast discrepancies in existing research (Simionato and Simpson, 2018).

From a clinical perspective, the overall problem of psychological health among mental health workers remains largely understudied. This topic is not only important for psychotherapists, but also for the clients who receive their help, as well as for the entire psychotherapeutic process. Thus, it is recommended that training programs for psychotherapists, within various therapeutic modalities, should include a greater focus on self-care behaviors among psychotherapists, to teach them to better manage their work-related distress and enhance their professional quality of life (McCormack et al., 2018). Further, current psychotherapists should continue to seek support within the profession as well as outside, in order to maintain their wellbeing and decrease the risk of experiencing burnout.

## Data Availability Statement

The original contributions presented in the study are included in the article/supplementary material, further inquiries can be directed to the corresponding author.

## Author Contributions

AV and MR contributed to conception and design of the study, performed the statistical analysis, and wrote sections of the manuscript. AV organized the database and wrote the first draft of the manuscript. Both authors contributed to manuscript revision, read, and approved the submitted version.

## Funding

This project has received funding from the New Ideas of POB V project implemented within the scope of the Excellence Initiative - Research University Program (Number PSP: 501-D125-20-5004310).

## Conflict of Interest

The authors declare that the research was conducted in the absence of any commercial or financial relationships that could be construed as a potential conflict of interest.

## Publisher's Note

All claims expressed in this article are solely those of the authors and do not necessarily represent those of their affiliated organizations, or those of the publisher, the editors and the reviewers. Any product that may be evaluated in this article, or claim that may be made by its manufacturer, is not guaranteed or endorsed by the publisher.
